# A Rare Case of Hyperammonemia Caused by Urinary Tract Infection Due to Corynebacterium riegelii

**DOI:** 10.7759/cureus.54082

**Published:** 2024-02-12

**Authors:** Kosuke Yada

**Affiliations:** 1 Internal Medicine, Chubu Rosai Hospital, Nagoya, JPN

**Keywords:** corynebacterium spp, corynebacterium riegelii, corynebacterium, hyperammonemia-encephalopathy, non-cirrhotic hyperammonemia

## Abstract

Hyperammonemia is a crucial differential diagnosis leading to consciousness disorders. While it is often associated with liver failure in most cases, it is imperative to be aware that hyperammonemia can also be induced by rare urease-producing bacteria, such as *Corynebacterium riegelii* causing obstructive urinary tract infections, as seen in this case. We report the case of an 85-year-old woman with no history of liver dysfunction and no previous indications of voiding difficulties. Based on the symptoms of consciousness impairment, elevated ammonia (NH_3_) levels in blood tests, and CT and urine findings, the diagnosis was obstructive uropathy due to urease-producing bacteria. Subsequent urine culture detected *Corynebacterium riegelii*, a urease-producing bacterium with limited reported cases. Treatment involved bladder catheterization and antibiotic administration, leading to a rapid improvement in consciousness. Given that this case, where voiding difficulties have not been previously diagnosed, exists, addressing voiding dysfunction is also crucial.

## Introduction

Considering hyperammonemia as a cause of disorders of consciousness is essential. While hepatic dysfunction is a common cause of hyperammonemia, other known etiologies include drug-induced factors (such as anti-cancer agents or valproic acid), urea cycle disorders, portosystemic shunting, and gastrointestinal bleeding, as well as obstructive urinary tract infection caused by urease-producing bacteria [1].

Obstructive urinary tract infection due to urease-producing bacteria is a well-known cause of hyperammonemia [2,3]. However, as seen in cases like the present one, hyperammonemia resulting from obstructive urinary tract infection due to *Corynebacterium riegelii* is extremely rare. The case of an 85-year-old woman, caused by this bacterium, is presented as follows.

## Case presentation

An 85-year-old woman with a medical history of rheumatoid arthritis and Sjogren's syndrome presented to the emergency department with a disturbance of consciousness. she had never been previously diagnosed with urinary retention or hepatic disorders before this hospitalization. She denied alcohol consumption and smoking. Upon examination, her temperature was 36.6℃, blood pressure was 136/80 mmHg, heart rate was 75 beats/minute, respiratory rate was 20/min, and oxygen saturation was 96% on room air. However, the Glasgow Coma Scale was 9. Pupils measured 3 mm/3 mm, and the pupillary light reflex was brisk. No nuchal rigidity was observed, and the upper limbs could not be raised, precluding the assessment of asterixis.

**Figure 1:**
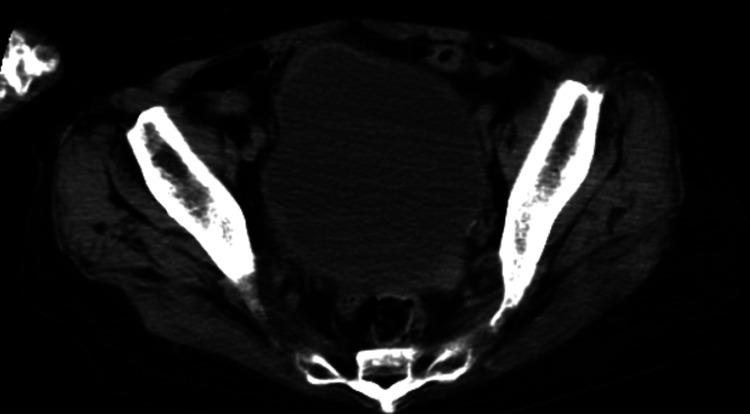
Plain CT of the lower abdomen The plain CT showed bladder distention.

**Figure 2:**
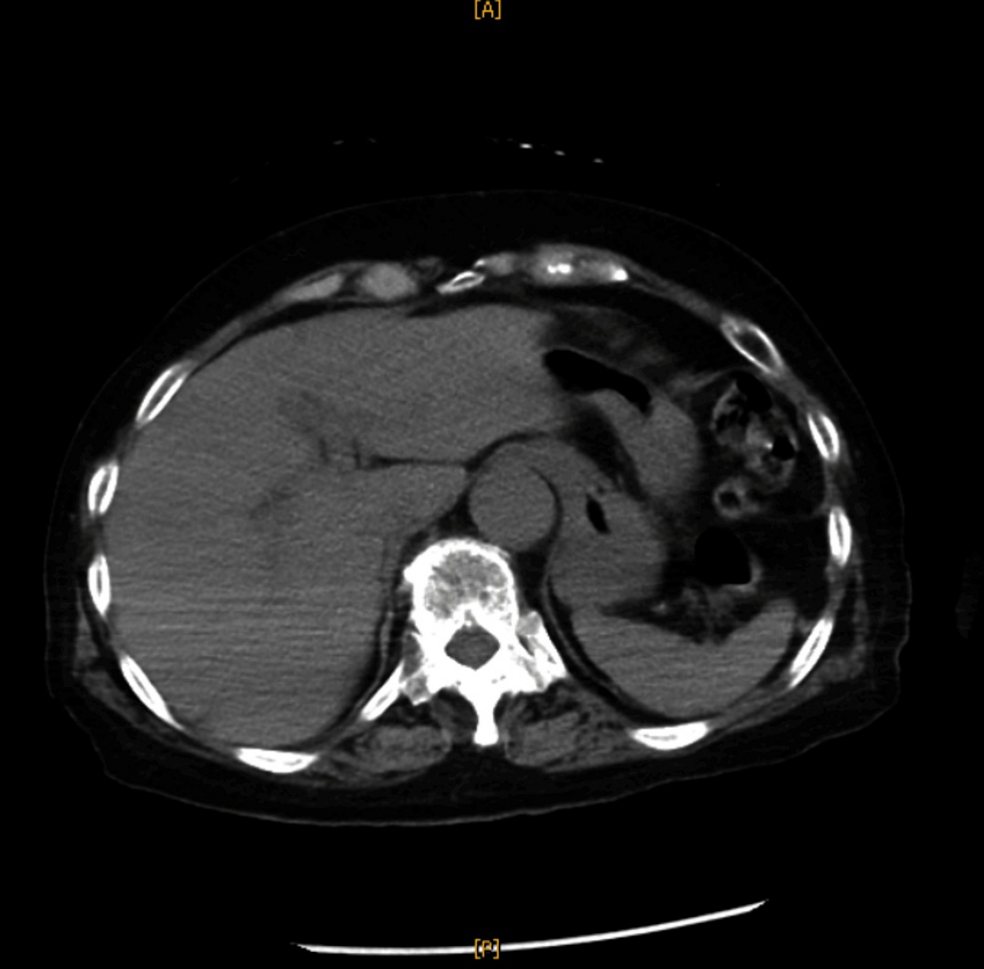
Plain CT of the upper abdomen There was no abnormality in the patient's liver.

**Figure 3:**
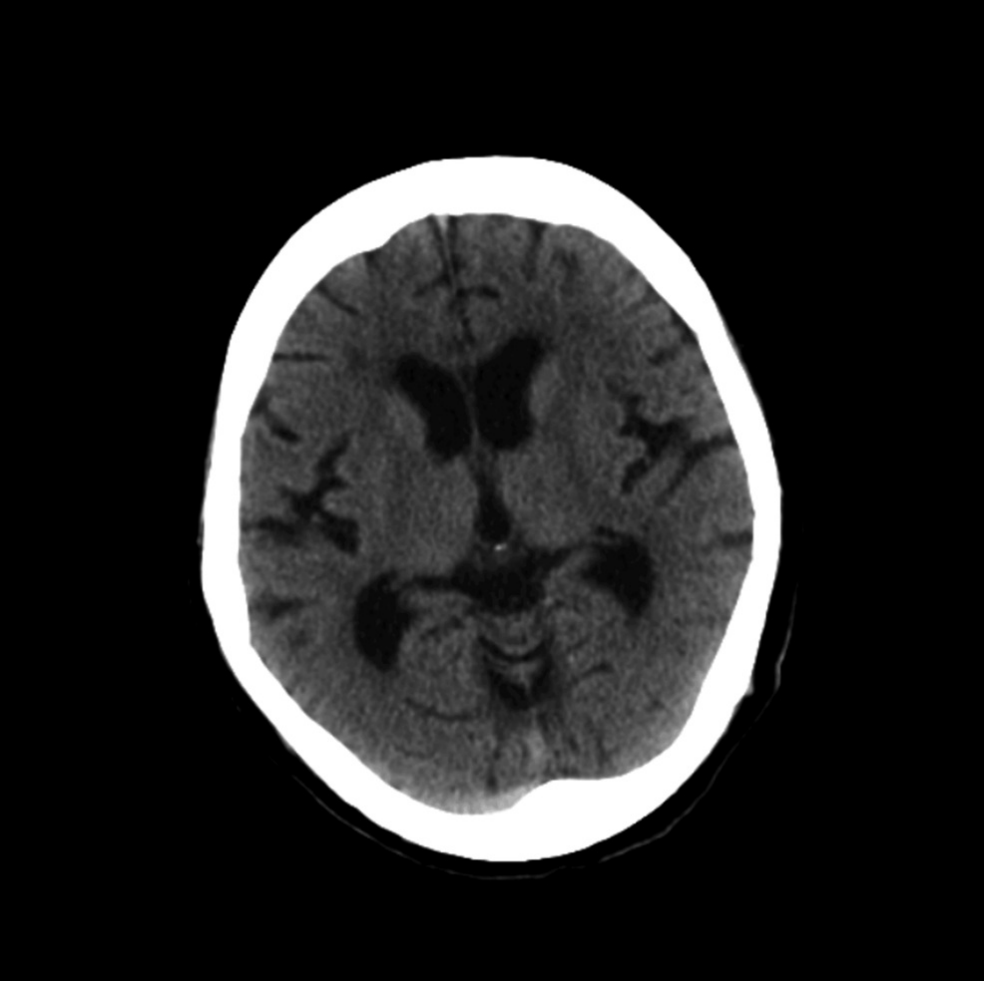
Plain CT of the head There was no abnormality.

Laboratory findings revealed a leukocyte count of 11,700/µL (normal range: 4,000-9,000 /μL) and C-reactive protein concentration of 2.94 mg/dL (normal range: < 0.3 mg/dL). Although ammonia (NH_3_) was 152 µg/dL (normal range: 15-45 μg/dL), aspartate aminotransferase (AST) concentration was 23 IU/L (normal range: 12-35 IU/L), and alanine transaminase (ALT) concentration was 10 IU/L (normal range: 6-40 IU/L). Urinalysis showed a urine pH of 8.5, urinary white blood cells 1+, and ammonium phosphate crystals +. CT showed significant bladder distension (Figure 1) but didn't show hepatic atrophy (Figure 2) and intracranial lesions causing consciousness impairment (Figure 3). *Corynebacterium riegelii* was detected in a urine culture. On the other hand, blood cultures were negative.

Considering these findings, the cause of the consciousness disorder was attributed to hyperammonemia. The elevated NH_3_ levels were thought to be associated with an increased urine pH, the presence of ammonium phosphate crystals, and bladder distension on CT, leading to the diagnosis of obstructive uropathy due to the urease-producing bacterium.

Initial treatment involved the placement of a bladder catheter and administration of the antibiotic ampicillin-sulbactam for a week. Consciousness improved within a few hours of catheter placement. However, on the third day, a recurrence of consciousness disorder occurred, revealing catheter obstruction. Prompt resolution of the obstruction resulted in a subsequent improvement in consciousness. Subsequent urological evaluation diagnosed neurogenic bladder, although the specific cause remained undetermined. Since prompt improvement in consciousness occurred after the initial treatment, MRI and cerebrospinal fluid analysis were not conducted.

## Discussion

Differential diagnoses for hyperammonemia include liver dysfunction, drug-induced causes (such as chemotherapy or valproic acid), urea cycle abnormalities, portosystemic shunts, and gastrointestinal bleeding [1]. Liver dysfunction is particularly common as an underlying cause. However, it is crucial to consider obstructive uropathy due to urease-producing bacteria, as seen in this case. Key findings, especially during an initial evaluation, include bladder distension on CT examination, elevated NH_3_ levels in blood tests, and the presence of high urinary pH and ammonium phosphate crystals in urine analysis.

Hyperammonemia leading to encephalopathy is likely to occur easily. Mechanisms of hyperammonemia due to urinary tract infections involve urease-producing bacteria. When these bacteria cause a urinary tract infection, urea is hydrolyzed by urease, producing NH_3_ in the bladder. Concurrent obstructive uropathy leads to increased bladder pressure, bladder overdistension, and enhanced absorption of NH_3_ into the bladder venous plexus. Since the bladder venous plexus directly drains into the inferior vena cava through the internal iliac veins, NH_3_ bypasses the portal circulation and undergoes hepatic metabolism less [4].

Urease-producing bacteria include *Corynebacterium *species, *Proteus *species, *Pseudomonas *species, and *Klebsiella *species [5]*. Corynebacterium urealyticum* is frequently reported in this context [6,7]. It is known for its resistance to many antibiotics, including beta-lactams, and susceptibility to glycopeptide antibiotics like vancomycin and teicoplanin [8]. On the other hand, reports on *Corynebacterium riegelii* are considerably rare. The strain in this case demonstrated susceptibility to a broad range of antibiotics, including beta-lactams, but further accumulation of cases is necessitated for comprehensive understanding.

In addition, in this case, urinary retention had not been diagnosed before. When managing obstructive urinary tract infections, it is crucial to evaluate whether urinary obstruction exists or not. This is because it can lead to preventing potential recurrence.

## Conclusions

In the differential diagnosis of confusion, it is crucial to consider hyperammonemia as a potential cause. Moreover, understanding the presence of obstructive uropathy due to urease-producing bacteria as a cause of hyperammonemia is essential. Key diagnostic steps during the initial consultation include urine analysis revealing high urinary pH and positive ammonium phosphate crystals, as well as confirming bladder distension through CT imaging. It is important to be aware that rare bacteria such as *Corynebacterium riegelii*, as seen in this case, could potentially contribute to the condition. Additionally, when encountering obstructive urinary tract infections without prior indications of voiding dysfunction, it is imperative to conduct a thorough assessment, investigate the underlying causes, and implement necessary treatment interventions.
